# A Brief History and the Current State of Robotic Mastectomy: a Review

**DOI:** 10.1007/s12609-025-00587-0

**Published:** 2025-07-11

**Authors:** Nicole Rademacher, Lauren A. Curwick, Catherine C. Parker

**Affiliations:** 1 Department of Surgery, University of Alabama at Birmingham, Birmingham, AL USA; 2 Division of Plastic Surgery, University of Alabama at Birmingham, Birmingham, AL USA; 3 Division of Breast and Endocrine Surgery, University of Alabama at Birmingham, 1808 7 th Avenue South, Boshell Diabetes Building, 512, Birmingham, AL 35233 USA

**Keywords:** Robotic mastectomy, Minimally invasive surgery, Nipple sparing mastectomy, Breast surgery, Breast cancer

## Abstract

**Purpose of Review:**

To evaluate and describe the current indications, implementation, operative techniques, and patient outcomes for robotic nipple sparing mastectomy (RNSM).

**Recent Findings:**

The robotic approach to nipple-sparing mastectomies (NSM) has been shown to be feasible. The learning curve required by surgical teams can be overcome, but barriers to implementation exist, including higher cost and longer operative time compared to conventional nipple sparing mastectomies (CNSM). When performed, RNSM have been found to confer greater patient satisfaction and similar if not improved perioperative outcomes. However, the most critical current concern is the lack of long-term oncologic outcomes. The current available literature suggests short-term oncologic outcomes are not significantly different between RNSM and CNSM.

**Summary:**

Randomized control trials with longer follow up are needed to determine the oncologic safety of RNSM and drive the future direction of this procedure.

## Introduction

Nipple-sparing mastectomy (NSM), in which the breast is removed but both the skin and nipple-areolar complex are retained, has previously been found to be an oncologically safe option for risk reduction and cancer treatment in select patients [1, 2]. Recent technological advances have led to the robotic nipple sparing mastectomy (RNSM). This was first described by Toesca et al. in 2015 for BRCA mutation carriers undergoing delayed contralateral risk-reducing NSM with immediate reconstruction [3]. The number of patients undergoing RNSM has grown largely due to its minimally invasive approach and smaller visible scar. Certain sites, notably Korea, Italy, Taiwan, and the United States have shed significant insight onto the procedure including feasibility, safety, and early outcomes data. However, appropriate use of RNSM remains controversial largely owing to the lack of long-term oncologic data [4–6]. Here, we aim to review the current indications, advantages and barriers, techniques, and outcomes of RNSM. Additionally, we summarize potential future directions for the procedure.

## Indications

Nipple sparing mastectomies have become widely acceptable for select patients, and NCCN guidelines state that “… NSM is probably equivalent to standard mastectomy [in regard to risk of locoregional cancer recurrence].” However, they do not comment on the use of the robotic approach [7]. The robotic approach has been used for both prophylactic and oncologic cases, but there are currently no evidence-based guidelines in practice regarding the use of RNSMs. In 2019, an international expert panel consisting of 10 surgeons met as part of International Endoscopic and Robotic Breast Surgery Symposium (IERBS) to create a consensus statement on robotic mastectomies [8]. The final consensus statement consisted of 53 individual statements with at least 80% agreement on each statement regarding indications and contraindications, technical considerations, patient counseling, outcome measures, and training and learning curve assessment. In summary, the indications for RNSM put forth by EIRBS include: moderate breast size, risk reducing mastectomies, and oncologic cases with tumor size ≤ 5 cm, favorable tumor biology, absence of NAC involvement, > 3 mm distance between tumor and skin, and low axillary disease burden [8]. There have been no additional consensus statements released since.

## Technique

The robotic approach to NSM has not been standardized, and the specific steps of a RNSM vary by institution and provider; however, broadly speaking there are common themes to the procedure. The patient is positioned supine on the operating table with the ipsilateral arm abducted. Depending on whether a single-port or multiport system is used will determine placement of the incision(s), but they most commonly are located on the lateral side of the breast near the axilla and easily concealed when a patient’s arms are at their sides. The main incision ranges from 2.5-6 cm, large enough to accommodate removal of the breast parenchyma and perform lymph node biopsy or dissection as indicated [3, 9–14]. A prospective study of 73 RNSM demonstrated an average incision length of 4 cm [15]. Some surgeons utilize a tumescent solution of saline with epinephrine with or without lidocaine to facilitate the dissection and reduce bleeding [9, 12, 14]. Next, a working space for port placement is created by dissecting the breast parenchyma anteriorly either sharply or with electrocautery. The robot is then docked, the port(s) are placed, and the breast is insufflated to 7-8 mmHg [3, 9–14]. The mastectomy dissection is continued and parenchyma is removed posteriorly off the pectoralis major, and the robotic arms can be used to retract and maintain exposure [3, 9–14]. Once the dissection is complete, the robot is undocked, the resected tissue is removed via the main incision, and a drain is placed in the resection bed. Immediate reconstruction is then performed with use of tissue expander, breast implant, or autologous flap [11].

The RNSM may be done with a multiport platform or a single-port platform. The multiport platform was used initially, but has notable disadvantages when compared to the single-port platform including increased arm collisions, ergonomic limitations, and decreased camera mobility with blind spots [16, 17]. The single-port approach has gained attention since first introduced in 2018 and has been used by multiple groups [16–18]. It too has limitations including decreased grasping power and greater difficulty creating counter retraction [17, 19]. The decision to use one system over the other depends largely on availability, patient needs and preferences, and surgeon experience and preference.

Both carbon dioxide insufflation and gasless approaches have been described in the literature for RNSM, though RNSM with insufflation are more prevalent. The gasless approach was designed to minimize the disadvantages of insufflation, such as obscured views from smoke, subcutaneous emphysema, and hypercapnia [14, 20]. However, gasless RNSM requires a longer incision and retraction with a self-retaining retractor that can lead to ischemic changes of the skin, causing some to abandon this approach altogether [19, 14]. A retrospective study comparing gasless to gas-inflated RNSM found that the incision was larger in the gasless group, but surgery time and complication rate and grade were not statistically different between the two groups [21].

Conversion to the open approach is rare for RNSM. In an early safety and feasibility study of 29 RNSM, 3 (6.9%) of cases were converted to open, due to extended duration of the operation, nipple-areolar positivity, and poor visualization around the curvature of the breast in the setting of the axillary incision being too posterior [22]. Another study evaluating 63 RNSM with immediate prosthetic breast reconstruction had a single case converted to open (1.6%) due to uncontrolled bleeding from an internal mammary perforator [9]. Other studies have cited a 0% conversion rate to open [16].

With the increase in interest in RNSM, immediate reconstruction using a robotic approach has followed suit. A commonly described technique is subpectoral implant reconstruction where, after completion of the mastectomy, a subpectoral space is developed under visualization with a lighted retractor before re-insufflation and a robotic approach is used for the remainder of the submuscular dissection. After completion of this dissection, the port is removed, and the axillary incision is used for implant insertion [3, 23]. Another technique described is a pre-pectoral implant where a robotic approach is used to secure the acellular dermal matrix around the breast pocket before manual insertion of the implant [24]. Like RNSM, immediate reconstruction using a robotic approach has not been widely implemented.

## Patient Outcomes

Some of the most cited advantages of RSNM are improved cosmesis and quality of life outcomes. RNSM offers a smaller incision than CNSM that can be easily hidden when patients have their arm at their side. A case–control comparison study found that RNSM with immediate gel implant breast reconstruction (IGBR) was associated with higher overall patient satisfaction as well as patient-reported esthetic outcomes as compared to CNSM with IGBR [23]. Similarly, a phase-III randomized control trial demonstrated that patients who underwent RNSM reported greater satisfaction with their breasts and well-being than those that underwent CNSM, and that their well-being remained at their pre-operative baseline whereas those that underwent CNSM were found to have a decline in well-being measures [25]. More recently, RNSM with IGBR and CNSM with IGBR were shown to confer similar breast and chest pain and upper extremity range of motion results [15].

Perioperative complications of NSM can include hematoma, seroma, and necrosis, with nipple necrosis being the most serious. Despite the increase in operative time, the data suggests there is no difference in number or type of complications with RNSM. A 2022 meta-analysis of 49 studies comparing complication rates of RNSM to CNSM found no difference in overall complication rates, rates of implant loss, hematoma or seroma formation, flap necrosis, or infection [26]. Another more recent meta-analysis of seven studies found reduced nipple necrosis in RNSM compared to CNSM, but similarly found no difference in grade III complications between the two groups [27]. Moreover, multiple studies have shown nipple necrosis rates to be lower in the RNSM group [28–30]. This is likely due to incision placement laterally and reduced tension on the skin itself.

Since a percentage of RNSM are performed for cancer, oncologic outcomes are important to evaluate. The rate of positive surgical margins has varied widely by study. An analysis of 22 patients that underwent RNSM with immediate breast reconstruction had no cases of positive surgical margins, and no local recurrences or deaths (mean follow up 6.9 ± 3.5 months) [12]. Other studies have found higher positive surgical margin rates from 2.6% to 16.7% [14, 31].

At present data is lacking to support the non-inferiority of RNSM compared to CNSM. The oncologic outcomes data currently available relies on short-term follow-up. An international multicenter survival analysis demonstrated no significant difference between RNSM and CNSM in disease free survival (DFS) (HR 0.19, 95% CI 0.001–1.58) and overall survival rates (HR 0.47, 95% CI 0.003–5.83), though median follow-up was only 18 months [30].

The risk of residual breast tissue (RBT) after NSM is of concern due to the possible association with recurrence. It is difficult to truly quantify RBT, but NSM is associated with more RBT than skin sparing mastectomy [32, 33], and since RNSM is associated with higher likelihood of preserved sensation, there is concern regarding the amount of RBT remaining [16]. A study evaluating cadaveric RNSM demonstrated flap thickness ranging from 2-3 mm with the most common location for RBT being the nipple-areolar complex [34]. Studies employing MRI to estimate RBT have demonstrated no difference in the rates of RBT in patients undergoing RNSM as compared to CNSM [35].

## Limitations of RNSM

Robotic surgery has been widely adopted in other areas of surgery, but significant barriers to its implementation exist. The learning curve required by surgical teams is a noted barrier to implementing RSNMs, but multiple groups have found that the duration of surgery decreases over time [9, 10, 14, 22]. Studies specifically aimed at analyzing the learning curve of a RNSM demonstrated that the number of cases needed to decrease operative time ranged from 2–3 to 27 demonstrating the variability in learning curves [19, 22, 36–38]. Learning curves likely depend on the surgeon’s robotic experience and comfort with the technology. However, a robotic approach is associated with higher operative time when compared to open approach even for experienced surgeons with operative time increasing by a mean difference of 58.81 min in meta-analysis data. [23, 25, 27]

Another barrier to the implementation of RNSM is cost. When taking into account the cost of the robotic system and maintenance, robotic surgeries have been found to be a more expensive option than its laparoscopic and open counterparts [39]. One case–control study out of Taiwan has shown RNSM with IGBR carries a higher financial burden than conventional nipple sparing mastectomy (CNSM) with IGBR (10,877 ± 796 versus 5,702 ± 661 US Dollars, *p* < 0.01) [23], and subsequent multicenter prospective study corroborated this data (10,301 ± 408 versus 6,254 ± 499 US Dollars for RNSM with IGBR and CNSM with IGBR respectively, *p* < 0.01) [15]. A meta-analysis that obtained cost data from three studies found RNSM was more expensive (median of 48.90 percent more expensive) than CNSM [27]. However, there remains a dearth of cost analyses comparing RNSMs to CNSMs. The RNSM has not been widely implemented, even at facilities that have the financial resources and experienced robotic teams to support such an endeavor. Ultimately, this is likely due to the lack of long-term patient outcomes data supporting its widespread implementation.

## Current State of the Robotic Nipple-Sparing Mastectomy and Future Direction

Despite advances in RNSM, the long-term oncologic outcomes remain unknown. Experts in the field of surgical breast oncology have expressed concerns regarding the oncologic outcomes and limited role of the RNSM [40]. Additionally FDA published 2 cautionary safety communications (2019 and again in 2021) warning patients of the lack of evidence of the safety and effectiveness of robotic mastectomies and that they had not yet received FDA authorization [41].

To address these concerns, there are ongoing clinical trials to further evaluate the safety and efficacy of RNSM. At present, there are several actively recruiting clinical trials [42] evaluating various outcome measures of RNSM with participating sites across the globe [43–47]. Study outcomes include adverse events, conversion rates, rates of positive surgical margins as well as oncologic outcomes including recurrence, disease-free survival, and overall survival.

## Conclusion

The robotic approach to NSMs has been shown to be a feasible approach and is associated with improved quality of life and patient satisfaction. Disadvantages of the procedure include cost, increased operative time, and need for additional skillset of the operative team. The most critical concern is whether the long-term oncologic outcomes of the minimally invasive robotic approach are equivalent to the conventional open approach. Until this data is available, the role of RNSM should be within the context of a clinical trial. In patients who are appropriate candidates for a NSM, the decision to pursue a minimally invasive approach through a clinical trial should be made through shared decision making between the patient and their surgeon considering the current outcomes evidence, surgeon and facility experience, and personal beliefs including quality of life discussions.

## Key References

- Toesca A, Sangalli C, Maisonneuve P, et al. A Randomized Trial of Robotic Mastectomy Versus Open Surgery in Women With Breast Cancer or BrCA Mutation. Annals of Surgery. 2022
  - ⚬ From the group that first described the RNSM, this randomized control trial showed Breast-Q scores were higher in the RNSM group than the CNSM group but no difference in complication number or type between the two groups.
- Lai HW, Chen DR, Liu LC, et al. Robotic Versus Conventional or Endoscopic-assisted Nipple-sparing Mastectomy and Immediate Prosthesis Breast Reconstruction in the Management of Breast Cancer: A Prospectively Designed Multicenter Trial Comparing Clinical Outcomes, Medical Cost, and Patient-reported Outcomes (RCENSM-P). Ann Surg. 2024;279(1):138–146. 10.1097/SLA.0000000000005924
  - ⚬ This recent (2024) multicenter study suggests RNSM has similar complications when compared to CNSM but remains a more expensive alternative.
- Park HS, Lee J, Lai HW, et al. Surgical and Oncologic Outcomes of Robotic and Conventional Nipple-Sparing Mastectomy with Immediate Reconstruction: International Multicenter Pooled Data Analysis. Ann Surg Oncol. 2022;29(11):6646–6657. 10.1245/s10434-022-11865-x
  - ⚬ This international multicenter pooled analysis demonstrated lower complication rates in patients who underwent RNSM compared to those who underwent CNSM and no statistically significant difference in early oncologic outcomes between the two groups.

## Data Availability

No datasets were generated or analysed during the current study.
